# A comparative study of different machine learning methods on microarray gene expression data

**DOI:** 10.1186/1471-2164-9-S1-S13

**Published:** 2008-03-20

**Authors:** Mehdi Pirooznia, Jack Y Yang, Mary Qu Yang, Youping Deng

**Affiliations:** 1 Department of Biological Sciences, University of Southern Mississippi, Hattiesburg, 39406, USA; 2 Harvard Medical School, Harvard University, Cambridge, Massachusetts 02140, USA; 3 National Human Genome Research Institute, National Institutes of Health (NIH), U.S. Department of Health and Human Services Bethesda, MD 20852, USA

## Abstract

**Background:**

Several classification and feature selection methods have been studied for the identification of differentially expressed genes in microarray data. Classification methods such as SVM, RBF Neural Nets, MLP Neural Nets, Bayesian, Decision Tree and Random Forrest methods have been used in recent studies. The accuracy of these methods has been calculated with validation methods such as v-fold validation. However there is lack of comparison between these methods to find a better framework for classification, clustering and analysis of microarray gene expression results.

**Results:**

In this study, we compared the efficiency of the classification methods including; SVM, RBF Neural Nets, MLP Neural Nets, Bayesian, Decision Tree and Random Forrest methods. The v-fold cross validation was used to calculate the accuracy of the classifiers. Some of the common clustering methods including K-means, DBC, and EM clustering were applied to the datasets and the efficiency of these methods have been analysed. Further the efficiency of the feature selection methods including support vector machine recursive feature elimination (SVM-RFE), Chi Squared, and CSF were compared. In each case these methods were applied to eight different binary (two class) microarray datasets. We evaluated the class prediction efficiency of each gene list in training and test cross-validation using supervised classifiers.

**Conclusions:**

We presented a study in which we compared some of the common used classification, clustering, and feature selection methods. We applied these methods to eight publicly available datasets, and compared how these methods performed in class prediction of test datasets. We reported that the choice of feature selection methods, the number of genes in the gene list, the number of cases (samples) substantially influence classification success. Based on features chosen by these methods, error rates and accuracy of several classification algorithms were obtained. Results revealed the importance of feature selection in accurately classifying new samples and how an integrated feature selection and classification algorithm is performing and is capable of identifying significant genes.

## Background

Microarray technology allows scientists to monitor the expression of genes on a genomic scale. It increases the possibility of cancer classification and diagnosis at the gene expression level. Several classification methods such as RBF Neural Nets, MLP Neural Nets, Bayesian, Decision Tree and Random Forrest methods have been used in recent studies for the identification of differentially expressed genes in microarray data. However there is lack of comparison between these methods to find a better framework for classification, clustering and analysis of microarray gene expression.

Another issue that might affect the outcome of the analysis is the huge number of genes included in the original data that some of them are irrelevant to analysis. Thus, reducing the number of genes by selecting those that are important is critical to improve the accuracy and speed of prediction systems. In this study, we compared the efficiency of the classification methods; SVM, RBF Neural Nets, MLP Neural Nets, Bayesian, Decision Tree and Random Forrest methods. We used v-fold cross validation methods to calculate the accuracy of the classifiers. We also applied some common clustering methods such as K-means, DBC, and EM clustering to our data and analysed the efficiency of these methods. Further we compared the efficiency of the feature selection methods; support vector machine recursive feature elimination (SVM-RFE) [1][2], Chi Squared [3], and CSF [4][5]. In each case these methods were applied to eight different binary (two class) microarray datasets. We evaluated the class prediction efficiency of each gene list in training and test cross-validation using our supervised classifiers. After features selection, their efficiencies are investigated by comparing error rate of classification algorithms applied to only these selected features versus all features.

### Supervised classification

Supervised classification, also called prediction or discrimination, involves developing algorithms to priori-defined categories. Algorithms are typically developed on a training dataset and then tested on an independent test data set to evaluate the accuracy of algorithms. Support vector machines are a group of related supervised learning methods used for classification and regression. The simplest type of support vector machines is linear classification which tries to draw a straight line that separates data with two dimensions. Many linear classifiers (also called hyperplanes) are able to separate the data. However, only one achieves maximum separation. Vapnik in 1963 proposed a linear classifier as a original optimal hyperplane algorithm [6]. The replacement of dot product by a non-linear kernel function allows the algorithm to fit the maximum-margin hyperplane in the transformed feature space [1-6]. SVM finds a linear separating hyperplane with the maximal margin in this higher dimensional space. $K\left(x_{i},x_{j}\right)=\Phi {\left(x_{i}\right)}^{T}\Phi \left(x_{j}\right)$ is called the kernel function [6]. There are four basic kernels: linear, polynomial, radial basic function (RBF), and sigmoid [7].

In decision tree structures, leaves represent classifications and branches represent conjunctions of features that lead to those classifications. There are advantages with decision tree algorithms: they are easily converted to a set of production rules, they can classify both categorical and numerical data, and there is no need to have a priori assumptions about the nature of the data. However multiple output attributes are not allowed in decision tree and algorithms are unstable. Slight variations in the training data can result it different attribute selections at each choice point within the tree. The effect can be significant since attribute choices affect all descendent subtrees [5]. ID3 (Iterative Dichotomiser 3) is an algorithm used to generate a decision tree. Developed by J. Ross Quinlan [8], ID3 is based on the Concept Learning System (CLS) algorithm [9]. J48 is an improved version of ID3 algorithm. It contains several improvements, including: choosing an appropriate attribute selection measure, handling training data with missing attribute values, handling attributes with differing costs, and handling continuous attributes [8].

Artificial Neural Networks (ANN) is an interconnected group of nodes that uses a computational model for information processing. It changes its structure based on external or internal information that flows through the network. ANN can be used to model a complex relationship between inputs and outputs and find patterns in data [10-12]. Two common ANN algorithms are Multi-layer perceptron (MLP) and Radial basis function (RBF) Networks (see methods) [13][14].

A bayesian network represents independencies over a set of variables in a given joint probability distribution (JPD). Nodes correspond to variables of interest, and arcs between two nodes represent statistical dependence between variables. Bayesian refers to Bayes' theorem on conditional probability. Bayes' theorem is a result in probability theory, which relates the conditional and marginal probability distributions of random variables. The probability of an event A conditional on another event B is in general different from the probability of B conditional on A. However, there is an explicit relationship between the two, and Bayes' theorem is the statement of that relationship [15]. Naive Bayes is a rule generator based on Bayes's rule of conditional probability. It uses all attributes and allows them to make contributions to the decision as if they were all equally important and independent of one another, with the probability denoted by the equation:

$$P\left(H|E\right)=\frac{P\left(E_{1}|H\right).P\left(E_{2}|H\right).....P\left(E_{n}|H\right)}{P\left(E\right)}$$

Where P(H) denotes the probability of event H, P(H|E) denotes the probability of event H conditional on event E, En is the n th attribute of the instance, H is the outcome in question, and E is the combination of all the attribute values [16].

Random forest is another classifier that consists of many decision trees. It outputs the class that is the mode of the classes output by individual trees [17][18]. Bagging (Bootstrap Aggregating) can also be used as an ensemble method [19] (see methods).

### Unsupervised clustering

Cluster-analysis algorithms group objects on the basis of some sort of similarity metric that is computed for features. Genes can be grouped into classes on the basis of the similarity in their expression profiles across tissues, cases or conditions. Clustering methods divide the objects into a predetermined number of groups in a manner that maximizes a specific function. Cluster analysis always produces clustering, but whether a pattern observed in the sample data remains an open question and should be answered by methods such as resampling-based methods. The k-means algorithm, Farthest First Traversal Algorithm, Density-based clustering, Expectation Maximization (EM) Clustering are four common methods used in this study [21-26].

### Feature selection

Feature selection methods can be divided into the *wrapper* model and the *filter* model [27]. The wrapper model uses the predictive accuracy of a mining algorithm to determine the goodness of a selected subset. Wrapper methods generally result in better performance than filter methods because the latter suffers from the potential drawback that the feature selection principle and the classification step do not necessarily optimize the same objective function [28]. In gene selection, the filter model is often adopted due to its computational efficiency [29]. Filter methods select predictive subset of the features using heuristics based on characteristics of the data. Moreover, in wrapper method, the repeated application of cross validation on the same data set might result in finding a feature subset that performs well on the validation data alone. Filter methods are much faster than wrapper methods and therefore are better suited to high dimensional data sets [30].

**SVM-RFE:** SVM-RFE is a feature selection method to filter out the optimum feature set by using SVM in a wrapper-style. It selects or omits dimensions of the data, depending on a performance measurement of the SVM classifier. One of the advantages of SVM-RFE is that it is much more robust to data overfitting than other methods [1]. This is an algorithm for selecting a subset of features for a particular learning task. The basic algorithm is the following: 1) Initialize the data set to contain all features, 2) Train an SVM on the data set, 3) Rank features according to c_i_ = (w_i_)^2^, 4) Eliminate the lower-ranked 50% of the features, 5) return to step 2. At each RFE step 4, a number of genes are discarded from the active variables of an SVM classification model. The features are eliminated according to a criterion related to their support for the discrimination function, and the SVM is re-trained at each step.

**Correlation based (CFS):** In CFS features can be classified into three disjoint categories, namely, strongly relevant, weakly relevant and irrelevant features [4][30]. Strong relevance of a feature indicates that the feature is always necessary for an optimal subset; it cannot be removed without affecting the original conditional class distribution. Weak relevance suggests that the feature is not always necessary but may become necessary for an optimal subset at certain conditions. Irrelevance indicates that the feature is not necessary at all. There are two types of measures for correlation between genes: linear and non-linear [4][29]. Linear correlation may not be able to capture correlations that are not linear. Therefore non-linear correlation measures often adopted for measurement. It is based on the information-theoretical concept of *entropy*, a measure of the uncertainty of a random variable [30,31].

**Chi Squared:** Another commonly used feature selection method is Chi-square statistic (χ^2^) method [3]. This method evaluates each gene individually by measuring the Chi-square statistics with respect to the classes. The gene expression numbers are first discretized into several intervals using an entropy-based discretization method. Then the Chi-square value of each gene is computed by

$$\chi^{2}=\sum_{i=1}^{m}\sum_{j=1}^{k}\frac{{\left(A_{ij}-\frac{R_{i}.C_{j}}{N}\right)}^{2}}{\frac{R_{i}.C_{j}}{N}}$$

Where *m* denotes the number of intervals, *k* the counts of classes, *N* the total number of patterns, *Ri* the number of patterns in the *i*th interval, *Cj* the number of patterns in the *j*th class, and *Aij* the number of patterns in the *i*th interval, *j*th class. The genes with larger Chi-square statistic values are then selected as marker genes for classification.

## Results and discussion

### Datasets

We applied classification, clustering, and feature selection methods to eight datasets in this work (Table 1). Each dataset is publicly available and data were downloaded from microarray repositories from caGEDA website from University of Pittsburgh [32]:

**Table 1:** Eight Datasets used in Experiment

**Dataset**	**Comparison**	**Variables (Genes)**	**Samples**
1. Lymphoma (Devos et.al, 2002)	Tumor vs. Normal	7129	25
2. Breast Cancer (Perou et. al, 2000)	Tumor subtype vs. Normal	1753	84
3. Colon Cancer (Alon et. al, 1999)	Epithelial vs. Tumor	7464	45
4. Lung Cancer (Garber et. al, 2001)	Tumor vs. Normal	917	72
5. Adenocarcinoma (Beer et.al, 2002)	NP vs. NN	5377	86
6. Lymphoma (Alizadeh et al, 2000)	DLBCL1 vs. DLBCL2	4027	96
7. Melanoma (Bittner et. al, 2000)	Tumor vs. Normal	8067	38
8. Ovarian Cancer (Welsh et. al, 2001)	Tumor vs. Normal	7129	39

▪ Lymphoma [33], contains 25 samples of which came from normal vs. malignant plasma cells including 7129 genes

▪ Breast Cancer [34], 84 samples of normal vs. tumor subtypes including 1753 genes

▪ Colon Cancer [35], 45 samples of Epithelial normal cells vs. tumor cells including 7464 genes

▪ Lung Cancer [36], contains 72 samples of which came from normal vs. malignant cells including 917 genes

▪ Adenocarcinoma [37], contains 86 samples of which came from survival in early-stage lung adenocarcinomas including 5377 genes

▪ Lymphoma [38], 96 samples of DLBCL1 vs. DLBCL2 cells including 4027 genes

▪ Melanoma [39], 38 samples of normal vs. malignant cells including 8067 genes

▪ Ovarian Cancer [40], 39 samples of normal vs. malignant cells including 7129 genes

### Pre-processing

We applied three steps pre-processing to the datasets. First we applied baseline shift for the datasets by shifting all measurements upwards by a number of means (or averages).

This process then followed by performing global mean adjustment. First, the global mean of all intensities of all datasets is calculated. Then, the difference between each individual mean and the global mean is calculated. This difference value is then added to (or subtracted from) each individual expression intensity value on each dataset. The result is that all datasets now have the same overall mean.

Finally a log transformation applied to the datasets. Log transformation has the advantage of producing a continuous spectrum of values.

### Classification

We used Weka [25] and SVM Classifier [7] for applying classification, clustering and feature selection methods to our datasets. In house java program was used to convert dataset from delimited file format, which is the default import format for SVM Classifier, to ARFF (Attribute-Relation File Format) file, the import format for Weka [25]. For the SVM we applied the following procedures.

First we transformed data to the format of the SVM software, ARFF for WEKA and Labeled them for SVM Classifier. Then we conducted simple scaling on the data. We applied linearly scaling each attribute to the range [-1, +1] or [0, 1].

We considered the RBF kernel and used cross-validation to find the best parameter C and γ. We used a “grid-search” [31] on C and γ using cross-validation. Basically pairs of (C, γ ) are tried and the one with the best cross-validation accuracy is picked. Trying exponentially growing sequences of C and γ is a practical method to identify good parameters [31], for example C = 2^-5^, 2^-3^, … , 2^15^ and γ = 2^-15^, 2^-13^, … , 2^3^.

**Figure 1:**
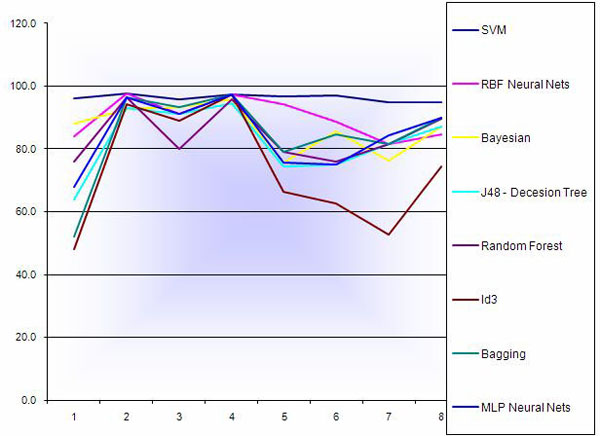
**Percentage accuracy of 10-fold cross validation of classification methods for all genes.** Results of 10-fold cross validation of the classification methods applied to all datasets without performing any feature selection.

The classification methods were first applied to all datasets without performing any feature selection. Results of 10-fold cross validation have been shown in Figure 1 and Table 2. In most datasets SVM and RBF neural nets performed better than other classification methods. In breast cancer data, SVM classification and RBF Neural Nets had the best accuracy 97.6%, and overall they performed very well on all datasets. The minimum accuracy for RBF we calculated was 81.6% over melanoma dataset. In lung cancer dataset MLP Neural Nets did also perform well and it was equal to SVM and RBF.

**Table 2:** Percentage accuracy of 10-fold cross validation of classification methods for all genes

**Dataset**	**SVM**	**RBF Neural Nets**	**MLP Neural Nets**	**Bayesian**	**J48 Decision Tree**	**Random Forest**	**Id3**	**Bagging**
1. Lymphoma (Devos et.al, 2002)	96.0	84.0	68.0	88.0	64.0	76.0	48.0	52.0
2. Breast Cancer (Perou et. al, 2000)	97.6	97.6	96.4	92.9	92.9	96.4	94.0	96.4
3. Colon Cancer (Alon et. al, 1999)	95.6	91.1	91.1	93.3	91.1	80.0	88.9	93.3
4. Lung Cancer (Garber et. al, 2001)	97.2	97.2	97.2	95.8	94.4	95.8	97.2	97.2
5. Adenocarcinoma (Beer et.al, 2002)	96.5	94.2	75.6	75.6	74.4	79.1	66.3	79.1
6. Lymphoma (Alizadeh et al, 2000)	96.9	88.5	75.0	85.4	75.0	76.0	62.5	84.4
7. Melanoma (Bittner et. al, 2000)	94.7	81.6	84.2	76.3	81.6	81.6	52.6	81.6
8. Ovarian Cancer (Welsh et. al, 2001)	94.9	84.6	89.7	87.2	87.2	89.7	74.4	89.7

The lowest accuracies are detected from Decision Tree algorithms (both J48 and ID3). As it is shown in Figure 1, in most cases they performed poorly comparing to other methods. Bayesian methods had also high accuracy in most datasets. Although it didn't performed as good as SVM and RBF, but the lowest accuracy was 85.4% on Lymphoma datasets. However overall we have to mention that it seems that in some cases performance of the classification methods depends on the dataset and a specific method cannot be concluded as a best method. For example Bayesian and J48 Decision Tree performed very well on colon and lung cancer, with 93% and 95% for Bayesian respectively and 91% and 94 % for J48, while RBF and MLP out performed on breast and lung cancer (97% and 96% respectively for MLP and 97% for both datasets for RBF).

**Figure 2:**
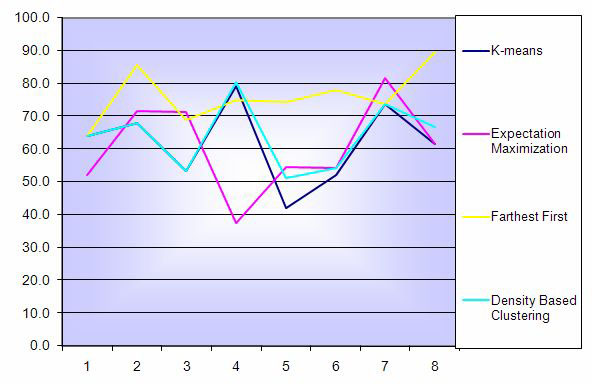
**Percentage accuracy of 10-fold cross validation of clustering methods for all genes**. Results of 10-fold cross validation of the two class clustering methods applied to all datasets,

We applied two class clustering methods to the datasets that are illustrated in Figure 1 and Table 3. As it is shown in Figures 2 we have a consistence performance of Farthest First in almost all datasets. EM performed poorly in Adenocarcinoma and Lymphoma datasets (54.7 and 54.2 respectively) while it was performing well in breast melanoma (81%).

**Figure 3:**
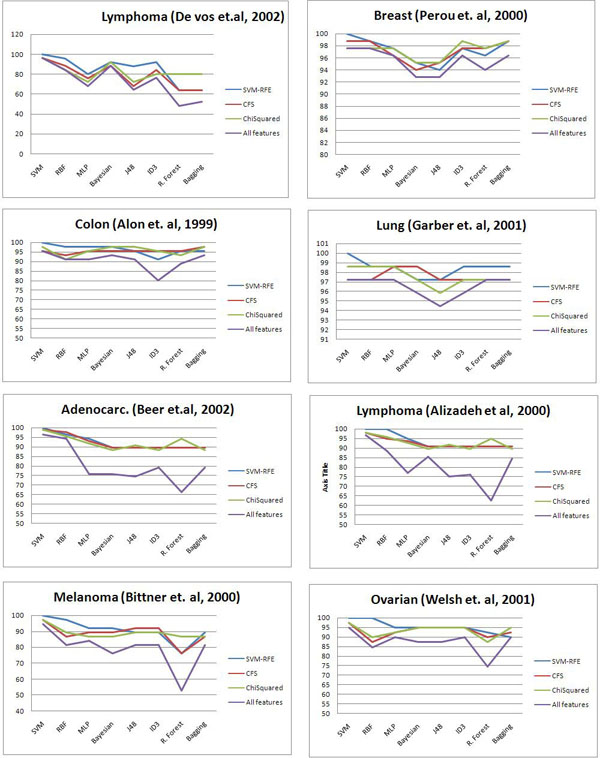
**Accuracy of 10-fold cross validation of feature selection and classification methods.** Accuracy of 10-fold cross validation of the pairwise combinations of the feature selection and classification methods

**Figure 4:**
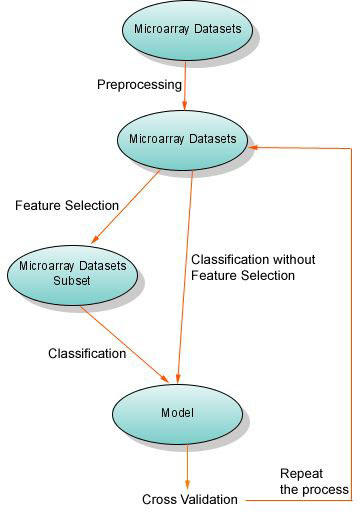
**Overview of the analysis pipeline**. The pipeline illustrates the procedure of the pairwise combinations of the feature selection and classification methods

**Table 3:** Percentage accuracy of 10-fold cross validation of clustering methods for all genes

**Dataset**	**K-means**	**Expectation Maximization**	**Farthest First**	**Density Based Clustering**
1. Lymphoma (Devos et.al, 2002)	64.0	52.0	64.0	64.0
2. Breast Cancer (Perou et. al, 2000)	67.9	71.4	85.7	67.9
3. Colon Cancer (Alon et. al, 1999)	53.3	71.1	68.9	53.3
4. Lung Cancer (Garber et. al, 2001)	79.2	37.5	75.0	80.6
5. Adenocarcinoma (Beer et.al, 2002)	42.0	54.7	74.4	51.2
6. Lymphoma (Alizadeh et al, 2000)	52.1	54.2	78.1	54.2
7. Melanoma (Bittner et. al, 2000)	73.7	81.6	73.7	73.7
8. Ovarian Cancer (Welsh et. al, 2001)	61.5	61.5	89.7	66.7

### The effect of feature selection

Pairwise combinations of the feature selection and classification methods were examined for different samples as it is shown in table 4 and 5 and Figure 1. The procedure is illustrated as a pipeline in Figure 1.

**Table 4:** 10-fold cross validation evaluation result of feature selection methods applied to the classification methods. X:Y pattern indicates X as the error rate in cancer samples and Y as the error rate in normal samples

**1. Lymphoma (De vos et.al, 2002)**	# Genes	SVM	RBF	MLP	Bayesian	J48	ID3	R. Forest	Bagging
SVM-RFE	50	0:0	0:1	2:3	1:1	1:2	1:1	4:5	3:6
CFS	50	1:0	2:1	3:3	2:1	4:4	2:2	3:6	3:6
ChiSquared	50	1:0	2:2	4:3	1:1	3:4	2:3	2:3	4:1
All features	7129	1:0	2:2	4:4	2:1	4:5	2:4	7:6	9:3
**2. Breast (Perou et. al, 2000)**	# Genes	SVM	RBF	MLP	Bayesian	J48	ID3	R. Forest	Bagging
SVM-RFE	50	0:0	1:0	1:1	3:1	4:1	1:1	2:1	1:0
CFS	50	1:0	1:0	2:1	3:2	3:1	1:1	1:1	1:0
ChiSquared	50	1:1	1:1	1:1	2:2	3:1	1:0	1:1	1:0
All features	1753	1:1	1:1	2:1	4:2	4:2	2:1	4:1	2:1
**3. Colon (Alon et. al, 1999)**	# Genes	SVM	RBF	MLP	Bayesian	J48	ID3	R. Forest	Bagging
SVM-RFE	50	0:0	0:1	1:0	1:0	2:0	3:1	1:1	1:1
CFS	50	1:1	2:1	1:1	2:0	1:1	2:2	1:1	1:0
ChiSquared	50	1:0	2:2	2:0	1:0	1:0	1:1	2:1	1:0
All features	7464	2:0	2:2	2:2	3:0	2:2	6:3	3:2	2:1
**4. Lung (Garber et. al, 2001)**	# Genes	SVM	RBF	MLP	Bayesian	J48	ID3	R. Forest	Bagging
SVM-RFE	50	0:0	0:1	1:0	1:1	1:1	1:0	1:0	1:0
CFS	50	1:1	1:1	1:0	1:0	1:1	1:1	1:1	1:1
ChiSquared	50	1:0	1:0	1:0	1:1	2:1	2:0	1:1	1:1
All features	917	2:0	2:0	1:1	2:1	2:2	2:1	1:1	1:1
**5. Adenocarc. (Beer et.al, 2002)**	# Genes	SVM	RBF	MLP	Bayesian	J48	ID3	R. Forest	Bagging
SVM-RFE	50	0:0	2:1	2:3	4:5	4:5	3:6	4:5	3:6
CFS	50	1:0	1:1	3:3	3:6	3:6	3:6	3:6	3:6
ChiSquared	50	1:0	2:2	4:3	5:5	3:5	5:5	2:3	5:5
All features	5377	2:1	3:2	15:6	15:6	15:7	14:4	17:13	12:6
**6. Lymphoma (Alizadeh et al, 2000)**	# Genes	SVM	RBF	MLP	Bayesian	J48	ID3	R. Forest	Bagging
SVM-RFE	50	0:0	0:1	2:3	4:5	4:5	3:6	4:5	3:6
CFS	50	1:1	2:3	3:3	3:6	3:6	3:6	3:6	3:6
ChiSquared	50	1:1	2:2	4:3	5:5	3:5	5:5	2:3	5:5
All features	4027	2:1	9:2	15:7	12:2	14:10	16:7	21:15	12:3
**7. Melanoma (Bittner et. al, 2000)**	# Genes	SVM	RBF	MLP	Bayesian	J48	ID3	R. Forest	Bagging
SVM-RFE	50	0:0	0:1	2:1	2:1	3:1	3:1	4:5	3:1
CFS	50	1:0	2:3	2:2	2:2	2:1	2:1	3:6	3:2
ChiSquared	50	1:0	2:2	3:2	2:3	2:2	2:2	2:3	3:2
All features	8067	2:0	4:3	4:2	6:3	4:3	4:3	15:3	5:2
**8. Ovarian (Welsh et. al, 2001)**	# Genes	SVM	RBF	MLP	Bayesian	J48	ID3	R. Forest	Bagging
SVM-RFE	50	0:0	0:1	1:1	1:1	1:1	1:1	2:1	3:1
CFS	50	1:0	3:2	1:2	1:1	1:1	1:1	2:2	2:1
ChiSquared	50	1:0	2:2	2:1	1:1	1:1	1:1	2:3	1:1
All features	7129	2:0	4:2	2:2	3:2	3:2	2:2	7:3	3:1

**Table 5:** Percentage accuracy of 10-fold cross validation of feature selection methods applied to the classification methods.

**1. Lymphoma (De vos et.al, 2002)**	# Genes	SVM	RBF	MLP	Bayesian	J48	ID3	R. Forest	Bagging
SVM-RFE	50	100.00	96.00	80.00	92.00	88.00	92.00	64.00	64.00
CFS	50	96.00	88.00	76.00	88.00	68.00	84.00	64.00	64.00
ChiSquared	50	96.00	84.00	72.00	92.00	72.00	80.00	80.00	80.00
All features	7129	96.00	84.00	68.00	88.00	64.00	76.00	48.00	52.00
**2. Breast (Perou et. al, 2000)**	# Genes	SVM	RBF	MLP	Bayesian	J48	ID3	R. Forest	Bagging
SVM-RFE	50	100.00	98.81	97.62	95.24	94.05	97.62	96.43	98.81
CFS	50	98.81	98.81	96.43	94.05	95.24	97.62	97.62	98.81
ChiSquared	50	97.62	97.62	97.62	95.24	95.24	98.81	97.62	98.81
All features	1753	97.62	97.62	96.43	92.86	92.86	96.43	94.05	96.43
**3. Colon (Alon et. al, 1999)**	# Genes	SVM	RBF	MLP	Bayesian	J48	ID3	R. Forest	Bagging
SVM-RFE	50	100.00	97.78	97.78	97.78	95.56	91.11	95.56	95.56
CFS	50	95.56	93.33	95.56	95.56	95.56	95.56	95.56	97.78
ChiSquared	50	97.78	91.11	95.56	97.78	97.78	95.56	93.33	97.78
All features	7464	95.56	91.11	91.11	93.33	91.11	80.00	88.89	93.33
**4. Lung (Garber et. al, 2001)**	# Genes	SVM	RBF	MLP	Bayesian	J48	ID3	R. Forest	Bagging
SVM-RFE	50	100.00	98.61	98.61	97.22	97.22	98.61	98.61	98.61
CFS	50	97.22	97.22	98.61	98.61	97.22	97.22	97.22	97.22
ChiSquared	50	98.61	98.61	98.61	97.22	95.83	97.22	97.22	97.22
All features	917	97.22	97.22	97.22	95.83	94.44	95.83	97.22	97.22
**5. Adenocarc. (Beer et.al, 2002)**	# Genes	SVM	RBF	MLP	Bayesian	J48	ID3	R. Forest	Bagging
SVM-RFE	50	100.00	96.51	94.19	89.53	89.53	89.53	89.53	89.53
CFS	50	98.84	97.67	93.02	89.53	89.53	89.53	89.53	89.53
ChiSquared	50	98.84	95.35	91.86	88.37	90.70	88.37	94.19	88.37
All features	5377	96.51	94.19	75.58	75.58	74.42	79.07	66.28	79.07
**6. Lymphoma (Alizadeh et al, 2000)**	# Genes	SVM	RBF	MLP	Bayesian	J48	ID3	R. Forest	Bagging
SVM-RFE	50	100.00	100.00	94.79	90.63	90.63	90.63	90.63	90.63
CFS	50	97.92	94.79	93.75	90.63	90.63	90.63	90.63	90.63
ChiSquared	50	97.92	95.83	92.71	89.58	91.67	89.58	94.79	89.58
All features	4027	96.88	88.54	77.08	85.42	75.00	76.04	62.50	84.38
**7. Melanoma (Bittner et. al, 2000)**	# Genes	SVM	RBF	MLP	Bayesian	J48	ID3	R. Forest	Bagging
SVM-RFE	50	100.00	97.37	92.11	92.11	89.47	89.47	76.32	89.47
CFS	50	97.37	86.84	89.47	89.47	92.11	92.11	76.32	86.84
ChiSquared	50	97.37	89.47	86.84	86.84	89.47	89.47	86.84	86.84
All features	8067	94.74	81.58	84.21	76.32	81.58	81.58	52.63	81.58
**8. Ovarian (Welsh et. al, 2001)**	# Genes	SVM	RBF	MLP	Bayesian	J48	ID3	R. Forest	Bagging
SVM-RFE	50	100.00	100.00	94.87	94.87	94.87	94.87	92.31	89.74
CFS	50	97.44	87.18	92.31	94.87	94.87	94.87	89.74	92.31
ChiSquared	50	97.44	89.74	92.31	94.87	94.87	94.87	87.18	94.87
All features	7129	94.87	84.62	89.74	87.18	87.18	89.74	74.36	89.74

First we tested SVM-RFE, Correlation based, and Chi Squared methods on several gene numbers (500, 200, 100, and 50). Methods were mostly consistent when gene lists of the top genes 50, 100, or 200 were compared. We selected 50 genes because it performed well, consumed less processing time, and required less memory configurations comparing to others.

Almost in all cases, the accuracy performance classifiers were improved after applying feature selections methods to the datasets. In all cases SVM-RFE performed very well when it applied with SVM classification methods.

In lymphoma dataset SVM-RFE performed 100% in combination of SVM classification method. Bayesian classification method performed well for SVM-RFE and Chi Squared feature selection methods with 92% accuracy in both cases.

CFS and Chi Squared also improved the accuracy of the classification. In breast cancer dataset the least improvement is observed from applying Chi Squared feature selection methods with no improvement over SVM, RBF and J48 classification methods with 97%, 84%, and 95% respectively.

In ovarian cancer dataset all feature selection methods performed very close to each other. However the SVM-RFE had a slightly better performance comparing to other methods. We detected 100% accuracy with SVM-RFE feature selection with both SVM and RBF classification methods. We also observed high accuracies among MLP classification and all feature selection methods with 94%, 92%, and 92% for SVM-RFE, CFS, and Chi Squared respectively.

In lung cancer datasets we can observe high accuracy in Decision Tree classification methods (both J48 and ID3) with all feature selection methods.

Overall we have to repeat again that although it is obvious that applying feature selection method improves the accuracy and also particularly it reduces the processing time and memory usage, but finding the best combination of feature selection and classification method might vary in each case.

## Conclusions

The bioinformatics techniques studied in this paper are representative of general-purpose data-mining techniques. We presented an empirical study in which we compare some of the most commonly used classification, clustering, and feature selection methods. We apply these methods to eight publicly available datasets, and compare, how these methods perform in class prediction of test datasets. We report that the choice of feature selection method, the number of genes in the gene list, the number of cases (samples) and the noise in the dataset substantially influence classification success. Based on features chosen by these methods, error rates and accuracy of several classification algorithms were obtained. Results reveal the importance of feature selection in accurately classifying new samples. The integrated feature selection and classification algorithm is capable of identifying significant genes.

## Methods

**Multi-layer perceptron (MLP):** Error backpropagation neural network is a feedforward multilayer perceptron (MLP) that is applied in many fields due to its powerful and stable learning algorithm [13]. The neural network learns the training examples by adjusting the synaptic weight according to the error occurred on the output layer. The back-propagation algorithm has two main advantages: local for updating the synaptic weights and biases, and efficient for computing all the partial derivatives of the cost function with respect to these free parameters. A perceptron is a simple pattern classifier.

The weight-update rule in backpropagation algorithm is defined as follows:

$\Delta w_{ji}\left(n\right)=\alpha \Delta w_{ji}\left(n-1\right)+\eta \delta_{j}(n)y_{i}\left(n\right)$ where *w* is the weight update performed during the *n*th iteration through the main loop of the algorithm, η is a positive constant called the learning rate, δ is the error term associated with j, and 0≤ α <1 is a constant called the momentum [9][11,12].

**Radial basis function (RBF) networks:** RBF networks have 2 steps of processing. First, input is mapped in the hidden layer. The output layer is then a linear combination of hidden layer values representing mean predicted output. This output layer value is the same as a regression model in statistics [9]. The output layer, in classification problems, is usually a sigmoid function of a linear combination of hidden layer values. Performance in both cases is often improved by shrinkage techniques, also known as ridge regression in classical statistics and therefore smooth output functions in a Bayesian network.

Moody and Darken [14] have proposed a multi-phase approach to RBFNs. This multi-phase approach is straight-forward and is often reported to be much faster than, e.g., the backpropagation training of MLP. A possible problem of the approach is that the RBF uses clustering method (e.g., k-means) to define a number of centers in input space and the clustering method is completely unsupervised and does not take the given output information into account. Clustering methods usually try to minimize the mean distance between the centers they distribute and the given data which is only the input part of the training data. Therefore, the resulting distribution of RBF centers may be poor for the classification or regression problem.

**Support Vector Machines (SVM):** Given a training set of instance-label pairs ($x_{i},y_{i}$), i = 1,…, l where $x_{i}\in R^{n}$ and $y\in {\left\{1,-1\right\}}^{l}$, the support vector machines require the solution of the following optimization problem:

$$\begin{array}{l} \underset{\omega ,b,\xi }{\min}\frac{1}{2}\omega^{T}\omega +C\sum_{i=1}^{l}\xi_{i}\\ y_{i}(\omega^{T}\phi (x_{i})+b\geq 1-\xi_{i}\\ \xi_{i}\geq 0 \end{array}$$

SVM finds a linear separating hyperplane with the maximal margin in this higher dimensional space. C > 0 is the penalty parameter of the error term. $K(x_{i},x_{j})=\Phi {\left(x_{i}\right)}^{T}\Phi (x_{j})$ is called the kernel function [6]. Here there are four basic kernels: linear, polynomial, radial basic function (RBF), and sigmoid:

Linear: $K(x_{i},x_{j})={x_{i}}^{T}x_{j}$

Polynomial: $K(x_{i},x_{j})={\left(x_{i},x_{j}\right)}^{d}$

RBF: $K(x_{i},x_{j})=\exp(-\frac{||x_{i}-x_{j}|{|}^{2}}{2\sigma^{2}})$

Sigmoid: $K(x_{i},x_{j})=\tanh(k(x_{i}x_{j})+\vartheta )$

**The k-means:** The k-means algorithm takes a dataset and partitions it into *k* clusters, a user-defined value. Computationally, one may think of this method as a reverse method of analysis of variance (ANOVA). The algorithm starts with *k* random clusters, and then move objects between those clusters with the goal to 1) minimize variability within clusters and 2) maximize variability between clusters [21]. In other words, the similarity rules will apply maximally to the members of one cluster and minimally to members belonging to the rest of the clusters. The significance test in ANOVA evaluates the between group variability against the within-group variability when computing the significance test for the hypothesis that the means in the groups are different from each other. Usually, as the result of a *k*-means clustering analysis, the means for each cluster on each dimension would be examined to assess how distinct *k* clusters are. Obtaining very different means for most is perfect [22].

**Farthest First:** Farthest First Traversal Algorithm works as a fast simple approximate clustering model after Simple K-Means. To find *k* cluster centers, it randomly chooses one point as a first center, and then selects point with maximal min-distance to current centers as a next center [23].

**Density Based Clustering (DBC):** Density-based clustering has turned out to be one of the most successful traditional approaches to clustering. It can be extended to detect subspace clusters in high dimensional spaces. A cluster is defined as a maximal set of density-connected points. Correlation clusters are sets of points that fit to a common hyperplane of arbitrary dimensionality. Density-based clustering starts by estimating the density of each point to identify core, border and noise points. A core point is referred to as a point whose density is greater than a user-defined threshold. A noise point is referred to as a point whose density is less than a user-defined threshold. Noise points are usually discarded in the clustering process. A non-core, non-noise point is considered as a border point [24].

**Expectation Maximization (EM) clustering:** An expectation-maximization (EM) algorithm finds maximum likelihood estimates of parameters in probabilistic models. EM performs repeatedly between an expectation (E) step, an expectation of the likelihood of the observed variables, and maximization (M) step, which computes the maximum expected likelihood found on the E step. EM assigns a probability distribution to each instance which indicates the probability of it belonging to each of the clusters [25]. By cross validation, EM can decide how many clusters to create.

The goal of EM clustering is to estimate the means and standard deviations for each cluster so as to maximize the likelihood of the observed data. The results of EM clustering are different from those computed by k-means clustering [26]. K-means assigns observations to clusters to maximize the distances between clusters. The EM algorithm computes classification probabilities, not actual assignments of observations to clusters.

**Cross validation:** In order to perform to measure classification error, it is necessary to have test data samples independent of the learning dataset that was used to build a classifier. However, obtaining independent test data is difficult or expensive, and it is undesirable to hold back data from the learning dataset to use for a separate test because that weakens the learning dataset. V-fold cross validation technique performs independent tests without requiring separate test datasets and without reducing the data used to build the tree. The learning dataset is partitioned into some number of groups called “folds” [31]. The number of groups that the rows are partitioned into is the ‘V’ in *V-fold cross classification*. 10 is the recommended and default number for “V”. It is also possible to apply the *v-fold cross-validation* method to a range of numbers of clusters in *k*-means or *EM* clustering, and observe the resulting average distance of the observations from their cluster centers.

Leave-one-out cross-validation involves using a single observation from the original sample as the validation data, and the remaining observations as the training data. This is repeated such that each observation in the sample is used once as the validation data [31].

## Competing interests

Financial support from Mississippi Computational Biology Consortium (MCBC) and Mississippi Functional Genomics Networks (MFGN) is gratefully acknowledged. The authors declare that they have no competing interests.

## Authors' contributions

MP and YD initiated the project. MP procured the necessary data and software, carried out the analyses, analyzed the results and drafted the manuscript. YD directed the design of the project and data analysis. JYY and MQY gave suggestions and helped to revise the manuscript. All authors read and approved the final manuscript.
